# Supplementary material for: DySTrack – a modular smart microscopy tool for live tracking of dynamic samples on modern commercial microscopes
Source: J Cell Sci. 2026 May 27;139(21):jcs264728. doi: 10.1242/jcs.264728 (PMC13286349; doi:10.1242/jcs.264728)
Supplement: Supplementary information [file joces-139-264728-s1.pdf]

Table S1. Comparison of relevant automation tools

Tool	Microscope control interface	Primary language	Compatible Hardware	Works on common Core Facility scopes?	Image analysis integration	License	Sources
**DySTrack**	Vendor GUIs (Nikon NIS, ZEN Blue, ZEN Black)	Python	Nikon (NIS), Zeiss (ZEN Blue), Zeiss (ZEN Black), +extendable	Yes	Full python ecosystem (scikit-image, scipy, etc.)	Open source	This paper
**Nikon NIS JOBS**	NIS-Elements native	JOBS macros	Nikon (NIS)	Yes (Nikon only)	NIS GA3, limited python IDE within JOBS	Vendor	[1]
**Zeiss ZEN OAD**	ZEN Blue native	IronPython / .NET	Zeiss (ZEN Blue)	Yes (Zeiss only)	Zeiss OAD / IronPython within ZEN Blue	Vendor	[2]
**MyPiC**	ZEN Black + GUI	VBA	Zeiss (ZEN Black)	Yes (Zeiss only)	Simple built-in options, or via Windows registry	Open Source	[3]
**µManager**	GUI (ImageJ) + Java/C++ API	Java / C++	100s of devices (stages, lasers, cameras, etc.)	Rarely installed, not supported by most vendors	ImageJ, Java / Beanshell scripts, various plugins	Open source	[4]
**Pycro-Manager**	µManager	Python	Same as µManager	Same as µManager	Full python ecosystem	Open source	[5]
**pymmcore, pymmcore-plus**	µManager	Python	Same as µManager	Same as µManager	Full python ecosystem	Open source	[6, 7]

[1] https://www.nisoftware.net/NikonSaleApplication/Help/Docs-AR/eng_ar/ug.jobs.html

[2] https://github.com/zeiss-microscopy/OAD

[3] https://github.com/manerotoni/mypic

[4] https://micro-manager.org

[5] https://github.com/micro-manager/pycro-manager

[6] https://github.com/micro-manager/pymmcore

[7] https://pymmcore-plus.github.io/pymmcore-plus

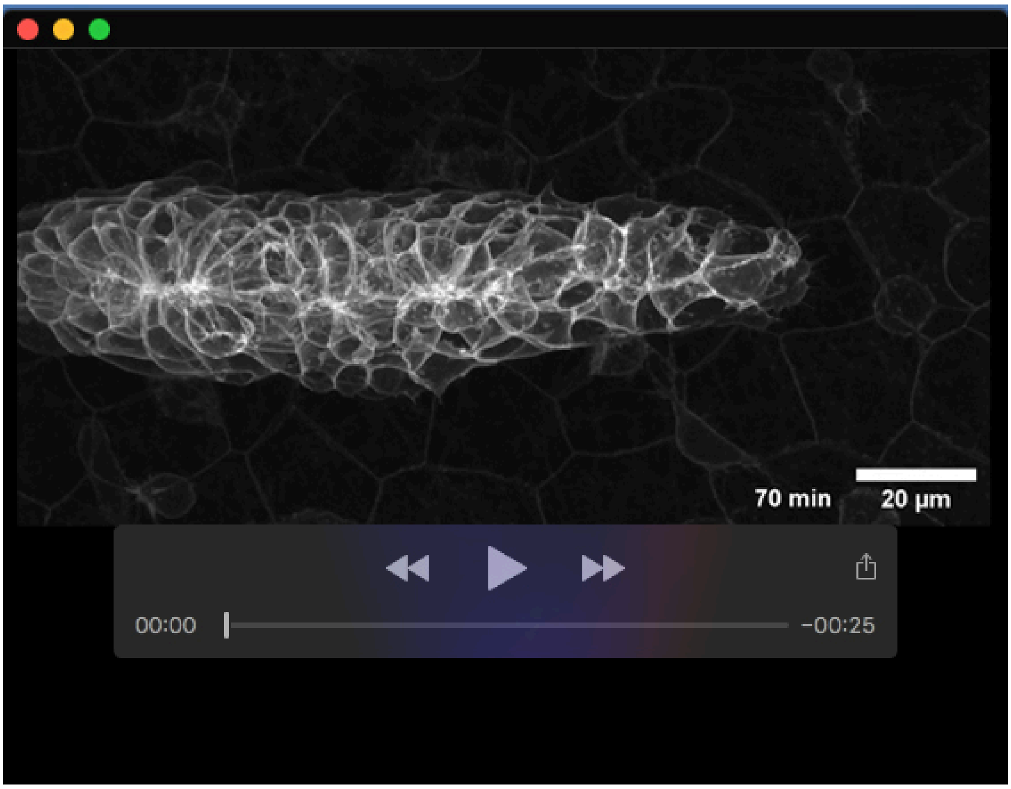

Movie 1. High-resolution tracked lateral line primordium

Maximum z-projected high-resolution (main scan) time course of a transgenically labeled pLLP (green; cldnB:lyn-EGFP) tracked with DySTrack on a Zeiss LSM880 in AiryScan FAST mode (40X 1.2NA water objective). Scale bar 20µm, time resolution 5min, and total time course duration 8.33h.

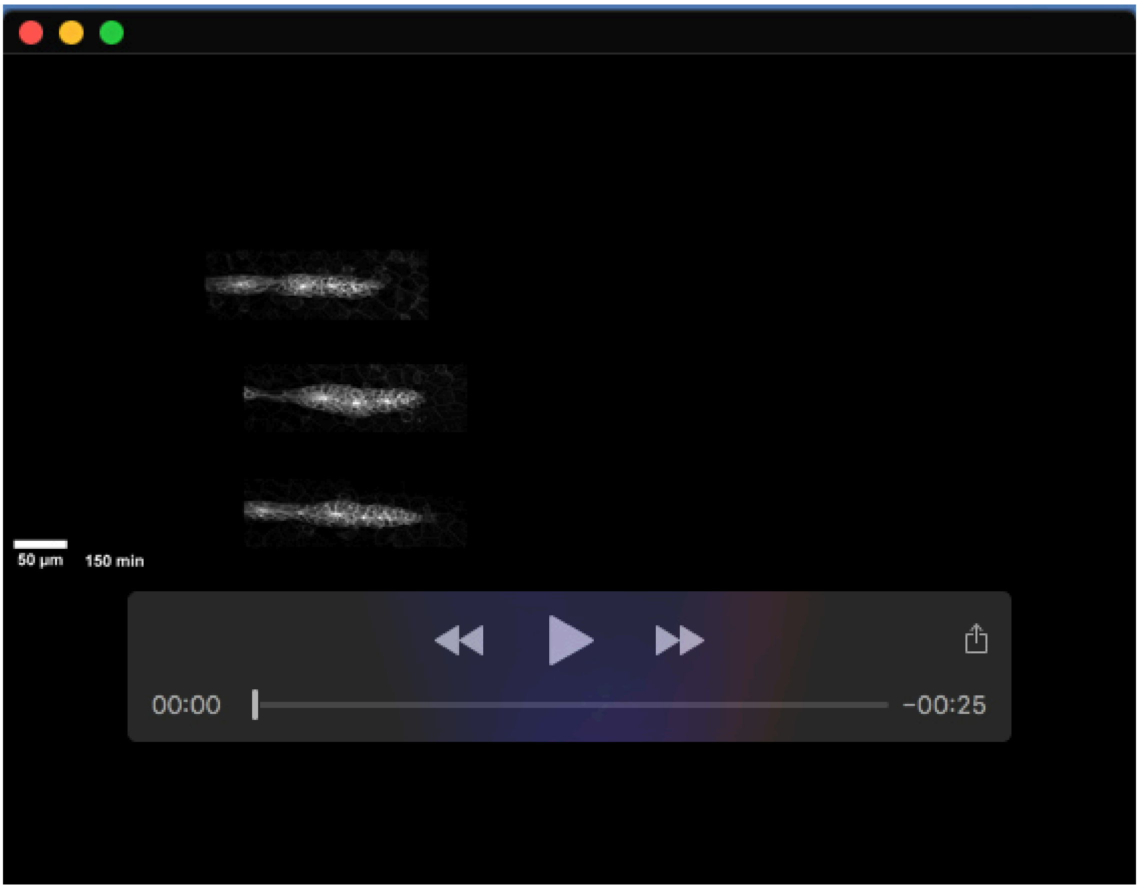

Movie 2. Multi-position pLLP acquisition with subsequent deregistration

Maximum z-projections of three simultaneously tracked lateral line primordia (green; cldnB:lyn-EGFP). The live registration applied by DySTrack was reverted *in post* to reconstruct the samples' actual movement in space (but note that positions in the y axis are not reflective of embryo locations in the dish). Raw data were acquired on a Zeiss LSM980 in AiryScan2 CO-8Y mode (40X 1.2NA water objective). Scale bar is 50µm, time resolution 10min, and total time course duration 14.66h. To cope with the size of this reconstruction, images were downsampled by 2x with bicubic interpolation.

Journal of Cell Science • Supplementary information

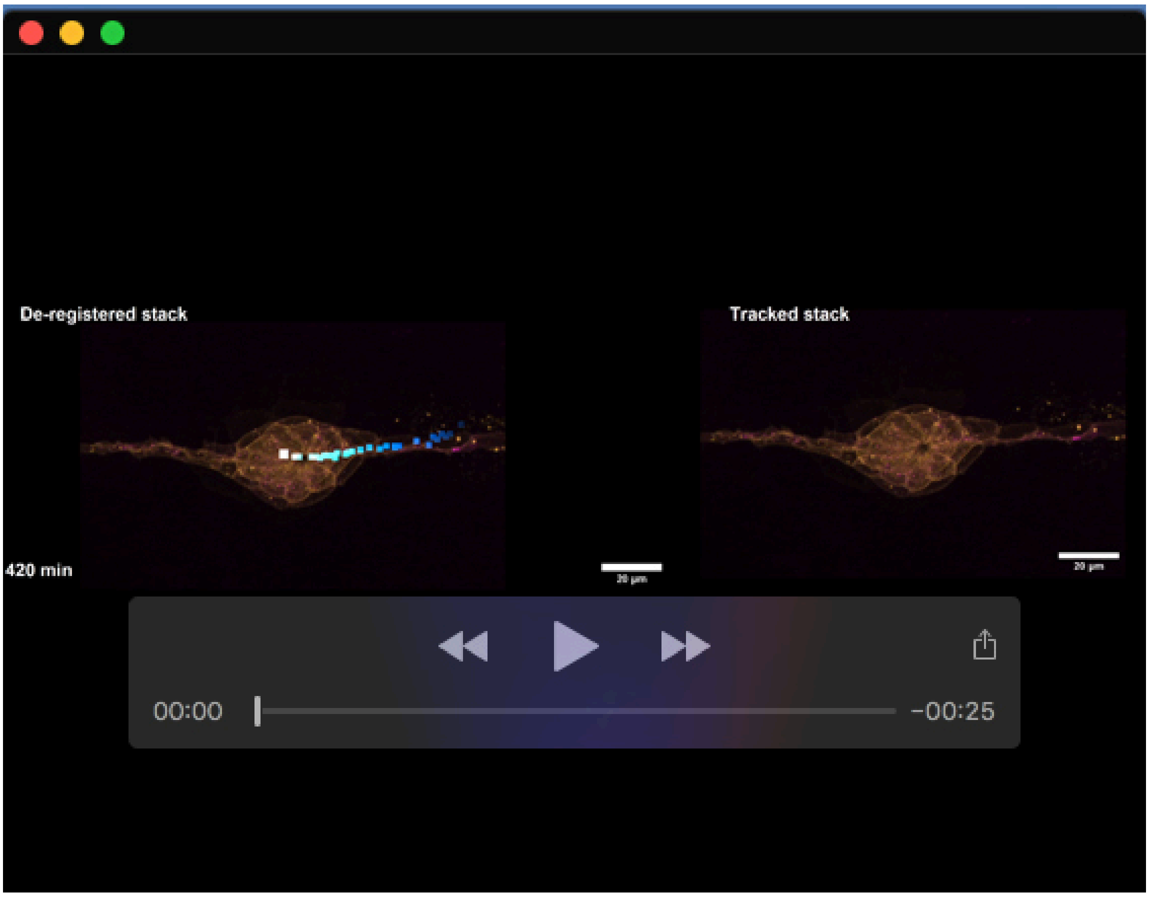

Movie 3. Long-term high-resolution tracking of a deposited neuromast

Maximum z-projections of a main scan time course of a deposited zebrafish neuromast, whose center of mass was tracked with DySTrack (right panel) based on the transgenic membrane label krt15:lyn-RFP (yellow). A second channel not used for tracking was also imaged, showing a transgenically labeled atypical chemokine receptor (cxcr7b:cxcr7b-EGFP; magenta). The movie was acquired on the Zeiss LSM980 in AiryScan2 CO-8Y mode (40X 1.2NA water objective). The live registration applied by DySTrack was reverted *in post* to reconstruct the sample's actual movement in space (de-registered stack; left panel) with the trace of DySTrack centroid positions indicated with white/cyan squares, revealing the sample drift that DySTrack has corrected. Scale bar is 20µm, time resolution 15min, and total time course duration 12h.

Journal of Cell Science • Supplementary information

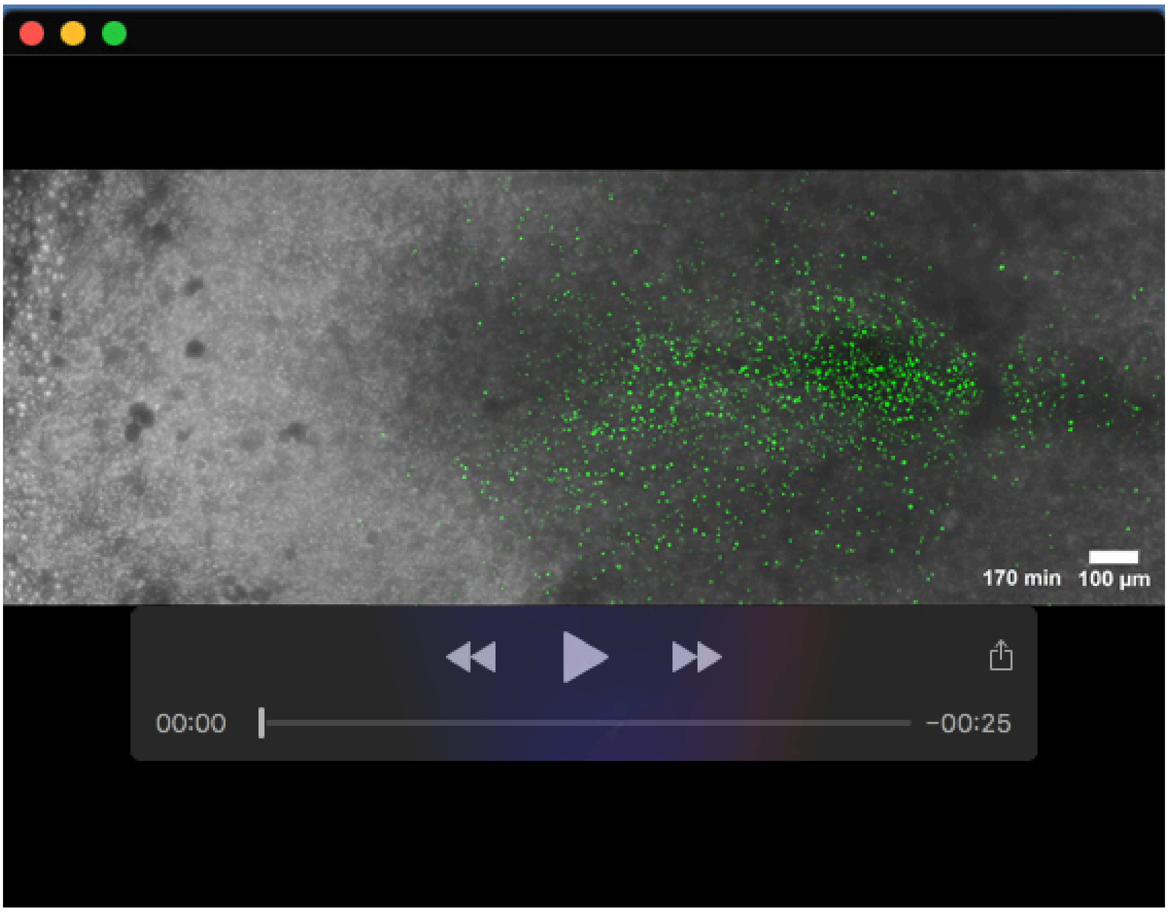

Movie 4. Multi-tile acquisition of tracked Hensen's node

Maximum z-projected main scan time course of the chick Hensen's node tracked with DySTrack on a Nikon AX R in resonant mode (20X 0.45NA 8.2mm-WD dry objective). Grayscale is transmission imaging, green is electroporated H2B-EGFP. The entire view is composed of three stitched acquisitions (15% overlap) with the right-most field of view being live tracked. The movie shows the node forming and regressing to the right (posterior) as somites are laid down. Note that the embryo begins to curve out of the plane in the second half of the movie, yet despite this issue – and despite changes in the morphology and labeling of the node – the model-based DySTrack pipeline is able to maintain stable tracking. Scale bar is 200µm, time resolution 10min, and total time course duration 22h.

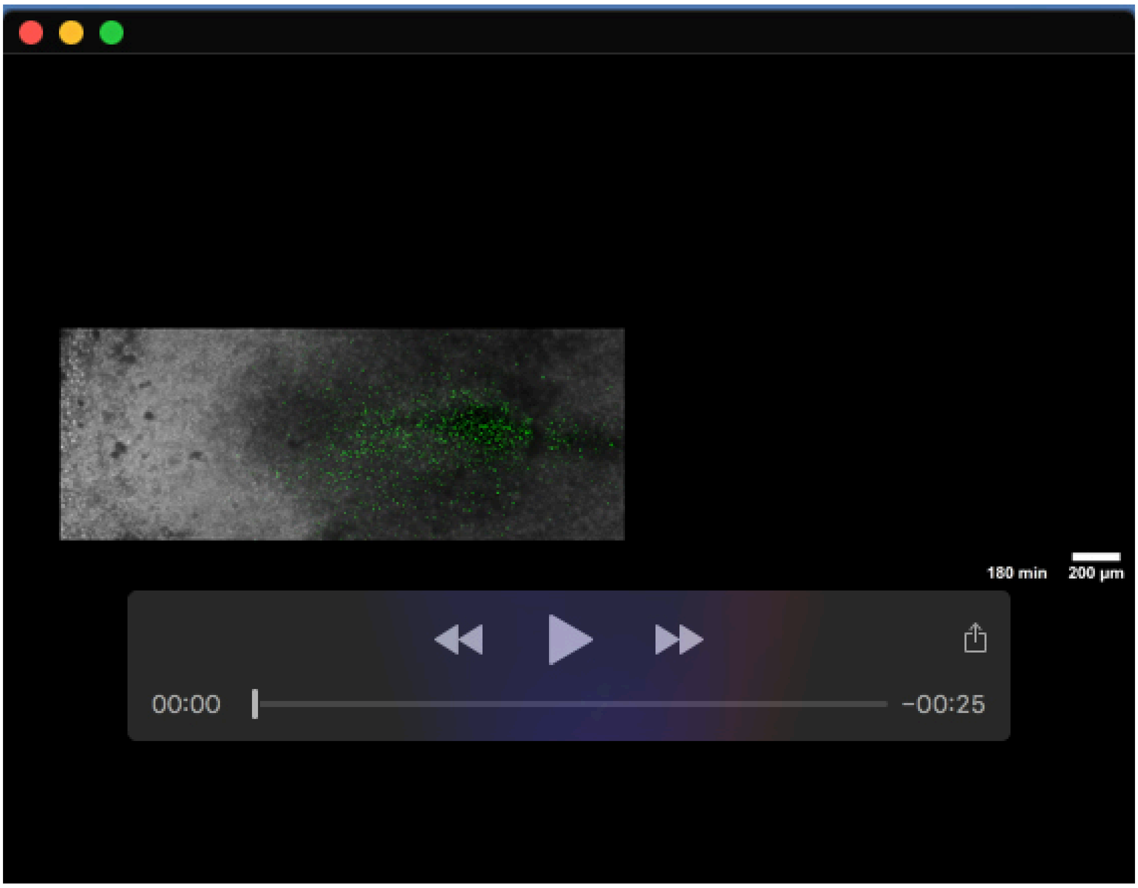

Movie 5. Deregistered tracked Hensen's node

The same data as shown in Supp. Movie 4, but deregistered *in post* to visualize the actual spatial displacement of the node during regression. Scale bar is 100µm, time resolution 10min, and total time course duration 22h. To cope with the size of this reconstruction, images were downsampled by 2x with bicubic interpolation.
