# Supplementary material for: DySTrack – a modular smart microscopy tool for live tracking of dynamic samples on modern commercial microscopes
Source: J Cell Sci. 2026 May 27;139(21):jcs264728. doi: 10.1242/jcs.264728 (PMC13286349; doi:10.1242/jcs.264728)
Supplement: Supplementary file 1 [file joces-139-264728_review_history.pdf]

DySTrack - a modular smart microscopy tool for live tracking of dynamic samples on modern commercial microscopes

Zimeng Wu, Octavian Voiculescu, Alessandro Mongera, Roberto Mayor, Mie Wong and Jonas Hartmann

DOI: 10.1242/jcs.264728

Editor: Guillaume Jacquemet

Review timeline

Original submission:	15 January 2026
Editorial decision:	2 March 2026
First revision received:	26 March 2026
Accepted:	18 April 2026

Original submission

First decision letter

MS ID#: jcs.264728

MS TITLE: DySTrack: a modular smart microscopy tool for live tracking of dynamic samples on modern commercial microscopes

AUTHORS: Zimeng Wu; Octavian Voiculescu; Alessandro Mongera; Roberto Mayor; Mie Wong; Jonas Hartmann
ARTICLE TYPE: Tools and Resources

Dear Dr Hartmann,

We have now reached a decision on the above manuscript.

As you will see, the reviewers raise a number of substantial criticisms that prevent me from accepting the paper at this stage. They suggest, however, that a revised version might prove acceptable, if you can address their concerns. If you think that you can deal satisfactorily with the criticisms on revision, I would be pleased to see a revised manuscript. We would then return it to the reviewers.

Reviewer 1: The authors describe Dystrack, a modular python software package to enable feedback microscopy on commercial systems (demonstrated in this manuscript on both Zeiss and Nikon hardware).

The authors correctly identify a major problem in so-called 'smart' microscopy, namely the bifurcation of this type of microscopy between standard hardware controls implemented by manufacturers, which are often limited, and more custom setups which require significant hardware and software engineering expertise to build and maintain. The manuscript presents a simple way to integrate arbitrary python image analysis code into a feedback microscopy setup, with the output being coordinates passed back to the microscopy control system to update the stage position based on the output of the image analysis script. While the overall approach is quite simple, consisting of three inter-operable scripts, this flexibility and simplicity is a core part of the appeal of this tool.

© 2026. Published by The Company of Biologists under the terms of the Creative Commons Attribution License (https://creativecommons.org/licenses/by/4.0/).

The paper is clearly written, simple to follow and all code is available and well documented, with examples. While I was not able to test the software on a physical microscope, I was able to install it, and tests resulted in the expected behavior ('saved' input image recognized, image analysis pipeline run, new coordinates written to file). I believe that this tool will be very valuable for the research community as a bridge between limited vendor supplied tools and the more complex custom microscopy setups. The two examples presented in the paper are compelling proof-of-concepts for tracking both a simple and a more complex (from an image analysis perspective) structure.

Several relatively minor additions could strengthen the manuscript:

- Given that DyStrack is suggested as a replacement for more rigid vendor-supplied tools built into microscope control software, a comparison with the best "state-of-the-art" approach implemented in the respective microscope control software (ie Nikon JOBS, etc) would be a useful demonstration of the increased utility of this tool over the currently existing situation.

- As the authors themselves note, and is specifically stated in their repository, there is some inherent danger in using such smart microscopy setups. In the materials and methods, the authors suggest some controls that were applied to, ie, limit the amount of stage movement. It would be helpful if a discussion of these safety measures could be added to the manuscript, as fear of hardware damage is likely to limit adoption of this tool.

Given the current state of smart microcopy adoption in research teams, I believe that an easy way to integrate arbitrary image analysis -pipelines into commercial microscope systems has the potential to be incredibly valuable to the research community, and that DyStrack fills a critical gap in the landscape, sitting in-between custom solutions based on, for example, micromanger, and vendor-supplied feedback microscopy tools.

However, the primary issue with this manuscript is that the guidelines for Tools and Resources papers indicate "Tools or techniques must also demonstrate proof of principle in the form of novel biological insight acquired using the method" (https://journals.biologists.com/jcs/pages/article-types#tools). This paper, unfortunately, while it does demonstrate convincingly the ability to successfully track moving tissues in developing embryos, does not include any such novel biological insight in either of the tissues studied.

As an aside, philosophically I believe that this requirement is unnecessary in general for tools and methods papers such as this. It is quite possible to demonstrate the utility of a tool to the field without contributing novel biological insight. This is particularly true in this case where the utility of an easy-to-implement smart-microscopy package to the field of cell and developmental biology is clear. However, short of advocating for a change in the journal editorial policies I unfortunately must conclude that this manuscript is not suitable for publication in JCS. If such a novel insight could be demonstrated, then given the potential for advancing smart microscopy in the cell and developmental biology community, this manuscript would be suitable for publication in JCS.

Reviewer 2: Review DySTrack

Tracking biological samples on the microscope is an experimental challenge scientists have been interested in for a long time (How to Track Bacteria, Berg, Howard C. 1971), even before the advent of digitally controlled microscopes. Surprisingly, this challenge hasn't become accessible even on modern microscopes, due to closed-source microscope control software and missing APIs, which make custom control of microscopes difficult and workflows hard to share across devices or labs.

The manuscript at hand describes a software tool that addresses this challenge and makes sample tracking accessible on many common microscope platforms. The article is well structured and easy to follow; the introduction provides good background on the promise of smart microscopes and the technical/systemic challenges that affect their development.

© 2026. Published by The Company of Biologists under the terms of the Creative Commons Attribution License (https://creativecommons.org/licenses/by/4.0/).

Relying on an interface layer that translates between the Python image analysis side and the different vendor microscope control software has many advantages compared to e.g. using MicroManager, which the authors list (not having to deal with low-level control, using the familiar GUI to set up the microscope configuration). However, this also comes with certain disadvantages that currently seem a bit missing from the manuscript. A short comment on the limitations of this type of setup versus e.g. using MicroManager + pycromanager/pymmcore would be appreciated. For example, while the authors claim that continued development will allow the software to be used for other use cases (line 215), it is unclear what the limitations are; the only example of data passed from the analysis script to the microscope are x/y/z positions. Would this system also be able to pass, for example, exposure times or commands to switch to a different imaging modality? As data is passed through files (or registry edits in the case of MyPic), how fast can files be written/read by the microscope control software? As there is no direct interface other than communication through files, how can they be synchronized, e.g. can the microscope wait for the next acquisition when the image processing function is not done yet? Are there potential race conditions, such as the microscope reading a half-written file? I'm not looking for a direct comparison between different software tools, just a short paragraph on the limitations aimed at potential users to help them understand potential constraints, and what expansions/customization options are in scope. Some of these points are already well addressed in the documentation.

While deep learning image analysis is ubiquitous in bioimage analysis, the authors show cleverly how classical image analysis methods can provide excellent results and are easily tunable to different use cases. This inspires confidence that labs trying to track different subjects, requiring custom image processing, would be able to adapt the software to their needs.

While I did not try to run the code myself, the GitHub repository is well documented and easy to navigate. The pipelines could be implemented as an abstract class with abstract methods to enforce adherence to the required inputs/outputs at a code level, or could contain a minimal example to use as a blueprint, but when consulting the documentation, the steps to implement a custom image processing function are very clear. Indeed, the documentation is very extensive, covers all microscopes that the manuscript claims compatibility with, and will be a very useful resource for users setting up the software, containing many practical tips for the programming as well as hardware side of things.

As a personal opinion, not required to be addressed in the revisions: the figures don't do justice to the rest of the manuscript/code/documentation. The movies are really impressive; I suggest trying to put one of these experiments in figure form into the first figure, to clearly show off the results your platform can achieve. While all information needed to understand the figures is provided in the legends, readers would benefit from adding more text directly into the figures (adding titles to graphs and images, labeling axes in kymographs). Also, in the kymograph, the scale bar of 3.92 h is unintuitive to interpret; a round number or better yet, adding y-ticks with time steps would help to also show the impressive duration the microscope was able to track the sample.

In summary, Wu et al. present a tool for robust tracking of diverse biological samples. They demonstrate long-term stability of the platform (some movies last 22 h!) on multiple common microscopy platforms. By expanding the functionality of vendor microscope control software, they provide an easy installation experience for new users. Together with the expansive documentation and well-maintained codebase, I'm confident that DySTrack will make smart tracking of live samples accessible to many new labs.

Minor text revisions requested:

- Discuss limitations of the architecture chosen; discuss scope of applications other than tracking

Other minor comments/questions:

- Line 142: "making the pLLP an ideal subject": the word "ideal" should be toned down a bit

- Line 322: "restrict stage movement to at most 1/10th of the depth": why is this? Same for line 346.

© 2026. Published by The Company of Biologists under the terms of the Creative Commons Attribution License (https://creativecommons.org/licenses/by/4.0/).

- How are pixel coordinates translated to stage coordinates? Are these values updated automatically when changing binning/objective magnification, etc.?

First revision

Author response to reviewers' comments

Point-by-point responses to reviewer comments

We thank both reviewers for thoroughly engaging with the manuscript and its associated code base, and for their encouraging and helpful comments, which allowed us to strengthen the paper through a number of targeted improvements. Our point-by-point responses are provided below (in blue). Line numbers noted here refer to the version of the revised manuscript in which changes are highlighted.

Reviewer #1

The authors describe Dystrack, a modular python software package to enable feedback microscopy on commercial systems (demonstrated in this manuscript on both Zeiss and Nikon hardware).

The authors correctly identify a major problem in so-called 'smart' microscopy, namely the bifurcation of this type of microscopy between standard hardware controls implemented by manufacturers, which are often limited, and more custom setups which require significant hardware and software engineering expertise to build and maintain. The manuscript presents a simple way to integrate arbitrary python image analysis code into a feedback microscopy setup, with the output being coordinates passed back to the microscopy control system to update the stage position based on the output of the image analysis script. While the overall approach is quite simple, consisting of three inter-operable scripts, this flexibility and simplicity is a core part of the appeal of this tool.

The paper is clearly written, simple to follow and all code is available and well documented, with examples. While I was not able to test the software on a physical microscope, I was able to install it, and tests resulted in the expected behavior ('saved' input image recognized, image analysis pipeline run, new coordinates written to file). I believe that this tool will be very valuable for the research community as a bridge between limited vendor supplied tools and the more complex custom microscopy setups. The two examples presented in the paper are compelling proof-of-concepts for tracking both a simple and a more complex (from an image analysis perspective) structure.

Several relatively minor additions could strengthen the manuscript:

- Given that DyStrack is suggested as a replacement for more rigid vendor-supplied tools built into microscope control software, a comparison with the best "state-of-the-art" approach implemented in the respective microscope control software (ie Nikon JOBS, etc) would be a useful demonstration of the increased utility of this tool over the currently existing situation.

We see DySTrack less as a replacement and more as an extension of vendor-supplied tools, but we certainly agree that there is value in further comparing and contrasting DySTrack to alternative means of implementing adaptive tracking. We now do so in an additional paragraph on this topic (lines 141ff.) and in a table that provides a comparative overview of key features/properties of the relevant frameworks (Supp. Table 1).

Direct comparisons to equivalent pipelines built entirely within vendor-supplied frameworks would in our view not add enough value to justify the required effort, especially considering that major factor in DySTrack's utility is its portability *across* microscopes, which such comparisons would not capture.

© 2026. Published by The Company of Biologists under the terms of the Creative Commons Attribution License (https://creativecommons.org/licenses/by/4.0/).

- As the authors themselves note, and is specifically stated in their repository, there is some inherent danger in using such smart microscopy setups. In the materials and methods, the authors suggest some controls that were applied to, ie, limit the amount of stage movement. It would be helpful if a discussion of these safety measures could be added to the manuscript, as fear of hardware damage is likely to limit adoption of this tool.

Thank you for raising this important point. We have now added an additional paragraph (lines 164ff.) discussing safety risks and mitigations.

Given the current state of smart microcopy adoption in research teams, I believe that an easy way to integrate arbitrary image analysis -pipelines into commercial microscope systems has the potential to be incredibly valuable to the research community, and that DyStrack fills a critical gap in the landscape, sitting in-between custom solutions based on, for example, micromanger, and vendor-supplied feedback microscopy tools.

However, the primary issue with this manuscript is that the guidelines for Tools and Resources papers indicate "Tools or techniques must also demonstrate proof of principle in the form of novel biological insight acquired using the method" (https://journals.biologists.com/jcs/pages/article-types#tools). This paper, unfortunately, while it does demonstrate convincingly the ability to successfully track moving tissues in developing embryos, does not include any such novel biological insight in either of the tissues studied.

As an aside, philosophically I believe that this requirement is unnecessary in general for tools and methods papers such as this. It is quite possible to demonstrate the utility of a tool to the field without contributing novel biological insight. This is particularly true in this case where the utility of an easy-to-implement smart-microscopy package to the field of cell and developmental biology is clear. However, short of advocating for a change in the journal editorial policies I unfortunately must conclude that this manuscript is not suitable for publication in JCS. If such a novel insight could be demonstrated, then given the potential for advancing smart microscopy in the cell and developmental biology community, this manuscript would be suitable for publication in JCS.

We thank the reviewer for recognizing and emphasizing the value of DySTrack to the field, and we agree that rigid application of the "novel insight" policy does not serve the community well. We have confirmed with the editor that in this particular case no further proof of principle is required.

Reviewer #2

Tracking biological samples on the microscope is an experimental challenge scientists have been interested in for a long time (How to Track Bacteria, Berg, Howard C. 1971), even before the advent of digitally controlled microscopes. Surprisingly, this challenge hasn't become accessible even on modern microscopes, due to closed-source microscope control software and missing APIs, which make custom control of microscopes difficult and workflows hard to share across devices or labs.

We now cite Berg's seminal paper in the introduction (lines 26f).

The manuscript at hand describes a software tool that addresses this challenge and makes sample tracking accessible on many common microscope platforms. The article is well structured and easy to follow; the introduction provides good background on the promise of smart microscopes and the technical/systemic challenges that affect their development.

Relying on an interface layer that translates between the Python image analysis side and the different vendor microscope control software has many advantages compared to e.g. using MicroManager, which the authors list (not having to deal with low-level control, using the familiar GUI to set up the microscope configuration). However, this also comes with certain disadvantages that currently seem a bit missing from the manuscript. A short comment on the limitations of this type of setup versus e.g. using MicroManager + pycromanager/pymmcore would be appreciated.

© 2026. Published by The Company of Biologists under the terms of the Creative Commons Attribution License (https://creativecommons.org/licenses/by/4.0/).

We agree that additional information on DySTrack's requirements, drawbacks and limitations is of value to readers. The revised manuscript mentions and discusses the requirements for vendor microscope control software (lines 100f.), the increased latency compared to vendor software workflows or µManager (lines 119f., lines 153f., line 268), and the specific focus of DySTrack on automated sample tracking as opposed to other smart microscopy applications (lines 155f.). Two of these points are part of a new paragraph that gives additional context on DySTrack's advantages and disadvantages (lines 141ff.), and also refers to a newly added table that provides a comparative overview of key features/properties of relevant frameworks (Supp. Table 1).

For example, while the authors claim that continued development will allow the software to be used for other use cases (line 215), it is unclear what the limitations are; the only example of data passed from the analysis script to the microscope are x/y/z positions. Would this system also be able to pass, for example, exposure times or commands to switch to a different imaging modality?

We have replaced the vague statement about other use cases (formerly line 215) with a new short paragraph (lines 276ff.) on our future plans for DySTrack, which are not focused on other use cases.

The limitation that DySTrack only passes x/y/z coordinates (and a short message) to the microscope is now discussed within the new paragraph on DySTrack's advantages and disadvantages mentioned (lines 155ff.). There, we also explain that simple flags can be passed via the message to e.g. switch imaging modality, and that additional data could be passed through separate files. However, we clarify that this requires customization and that other software tools may be more suitable for smart microscopy applications other than sample tracking.

As data is passed through files (or registry edits in the case of MyPic), how fast can files be written/read by the microscope control software? As there is no direct interface other than communication through files, how can they be synchronized, e.g. can the microscope wait for the next acquisition when the image processing function is not done yet?

As detailed above, we now note in several places that additional latency must be expected because of the file-based interface. Synchronization however is not an issue, as the microscope macros enter a (blocking) loop while awaiting new coordinates from DySTrack, so the microscope will always wait until the image processing function is done. This is now made explicit in the text (lines 88-89) as part of a few extra lines we added to elaborate on the functionality of the microscope macro (lines 85f.)

Are there potential race conditions, such as the microscope reading a half-written file?

This is an excellent point; thank you. We had considered the possibility of race conditions arising when the microscope control software writes out a (prescan) image file that is being awaited by DySTrack manager, and we provide a robust wrapper for image file reading to guard against this issue (now mentioned in line 112).

However, we had not previously considered the possibility of a race condition arising when DySTrack writes the new coordinates that are being awaited by the microscope macro, simply because very little data is being written. We have now formally tested this and found that, indeed, the small amount of data being written means that the write operation occurs in a single flush of the buffer, so there is no risk that the microscope might (attempt to) read incomplete coordinates (now stated in lines 133-134).

This could change if substantial amounts of additional data were "encoded" into the message column of the coordinate file. We therefore now recommend that additional data be sent to the microscope via a separate file (lines 157f.) and we have updated our documentation accordingly (cf. Issue #18, Release v1.0.2).

I'm not looking for a direct comparison between different software tools, just a short paragraph on the limitations aimed at potential users to help them understand potential constraints, and what expansions/customization options are in scope. Some of these points are already well addressed in the documentation.

© 2026. Published by The Company of Biologists under the terms of the Creative Commons Attribution License (https://creativecommons.org/licenses/by/4.0/).

We believe this has now been addressed through the updates and additions described above.

While deep learning image analysis is ubiquitous in bioimage analysis, the authors show cleverly how classical image analysis methods can provide excellent results and are easily tunable to different use cases. This inspires confidence that labs trying to track different subjects, requiring custom image processing, would be able to adapt the software to their needs.

While I did not try to run the code myself, the GitHub repository is well documented and easy to navigate. The pipelines could be implemented as an abstract class with abstract methods to enforce adherence to the required inputs/outputs at a code level, or could contain a minimal example to use as a blueprint, but when consulting the documentation, the steps to implement a custom image processing function are very clear. Indeed, the documentation is very extensive, covers all microscopes that the manuscript claims compatibility with, and will be a very useful resource for users setting up the software, containing many practical tips for the programming as well as hardware side of things.

We agree that taking an object-oriented rather than functional approach to DySTrack pipelines would offer some technical advantages. However, to our knowledge, object-oriented programming is not widely taught in introductory python and image analysis courses for bioscientists, so abstract classes are a feature that even relatively advanced users of DySTrack may never have encountered.

A minimal example of a pipeline function that can serve as an explainer and blueprint is a good idea; the `center_of_mass` pipeline was initially intended as such, but has since grown in complexity. We have now added a simplified and annotated version of that pipeline as a minimal example and have updated the documentation to refer to this example where appropriate (Issue #16, Release v1.0.2).

As a personal opinion, not required to be addressed in the revisions: the figures don't do justice to the rest of the manuscript/code/documentation. The movies are really impressive; I suggest trying to put one of these experiments in figure form into the first figure, to clearly show off the results your platform can achieve. While all information needed to understand the figures is provided in the legends, readers would benefit from adding more text directly into the figures (adding titles to graphs and images, labeling axes in kymographs). Also, in the kymograph, the scale bar of 3.92 h is unintuitive to interpret; a round number or better yet, adding y-ticks with time steps would help to also show the impressive duration the microscope was able to track the sample.

We thank the reviewer for this feedback, which prompted us to make a number of improvements. While we did not find an agreeable way of including one of our movies in the first figure, we now refer to Supp. Movies 1 and 2 in the introduction (line 54), prompting readers to view them early on. Furthermore, we have added various extra annotations to all figures and have replaced the vertical scale bar in Fig. 2d-e with y-ticks. Finally, we now include new Fig. 2f-g showing a 3D single-cell segmentation of the stack in Fig. 3c to illustrate that DySTrack main scans of the pLLP are of segmentable quality (discussed in the text in lines 216f.).

In summary, Wu et al. present a tool for robust tracking of diverse biological samples. They demonstrate long-term stability of the platform (some movies last 22 h!) on multiple common microscopy platforms. By expanding the functionality of vendor microscope control software, they provide an easy installation experience for new users. Together with the expansive documentation and well-maintained codebase, I'm confident that DySTrack will make smart tracking of live samples accessible to many new labs.

Minor text revisions requested:

- Discuss limitations of the architecture chosen; discuss scope of applications other than tracking

See our responses above for details on how we have addressed this point.

Other minor comments/questions:

- Line 142: "making the pLLP an ideal subject": the word "ideal" should be toned down a bit

© 2026. Published by The Company of Biologists under the terms of the Creative Commons Attribution License (https://creativecommons.org/licenses/by/4.0/).

Changed to "a well-suited subject".

- Line 322: "restrict stage movement to at most 1/10th of the depth": why is this? Same for line 346.

As we now discuss in a new paragraph (lines 164ff.), microscope automation always comes with some risk of damage. Since stage movement in z represents the most significant concern when using DySTrack, we introduce this limit into our pipelines to preclude sudden jumps due to unexpected errors (e.g. integer overflows). In practice, this is mainly relevant during early testing of new pipelines where potential bugs are yet to be discovered, but it doesn't hurt to keep the safety limit in place even in well-established pipelines.

- How are pixel coordinates translated to stage coordinates? Are these values updated automatically when changing binning/objective magnification, etc.?

Coordinates returned by our pipeline are typically in units pixel and in the image frame of reference. They are then converted within the microscope macro to units micron and to the stage frame of reference (now mentioned in lines 89f.). This is based on the acquisition settings and thus adapts completely automatically to changes in binning or objective magnification. The technical considerations behind implementing this are described in detail in the documentation (Running DySTrack > DySTrack on other microscopes > Notes on required features > Point 7).

<u>Second decision letter</u>

MS ID#: jcs.264728R1

MS Title: DySTrack: a modular smart microscopy tool for live tracking of dynamic samples on modern commercial microscopes

Authors: Zimeng Wu; Octavian Voiculescu; Alessandro Mongera; Roberto Mayor; Mie Wong; Jonas Hartmann
Article Type: Tools and Resources

Dear Dr Hartmann,

I am happy to tell you that your manuscript has been accepted for publication in Journal of Cell Science, pending standard publication integrity checks.

© 2026. Published by The Company of Biologists under the terms of the Creative Commons Attribution License (https://creativecommons.org/licenses/by/4.0/).
